# ChAdOx1 nCoV‐19 vaccination generates spike‐specific CD8^+^ T cells in aged mice

**DOI:** 10.1111/imcb.12645

**Published:** 2023-05-06

**Authors:** William S Foster, Joseph Newman, Nazia Thakur, Alexandra J Spencer, Sophie Davies, Danielle Woods, Leila Godfrey, Sarah H Ross, Hayley J Sharpe, Arianne C Richard, Dalan Bailey, Teresa Lambe, Michelle A Linterman

**Affiliations:** ^1^ Lymphocyte Signalling and Development Babraham Institute, Babraham Research Campus Cambridge UK; ^2^ The Pirbright Institute Pirbright, Woking UK; ^3^ The Jenner Institute, University of Oxford Oxford UK; ^4^ Oxford Vaccine Group, Medical Sciences Division, Department of Paediatrics University of Oxford and Chinese Academy of Medical Science (CAMS) Oxford Institute (COI), University of Oxford Oxford UK; ^5^ Signalling Programme, Babraham Institute, Babraham Research Campus Cambridge UK; ^6^ Present address: School of Biomedical Sciences and Pharmacy University of Newcastle Newcastle NSW Australia

**Keywords:** Aging, CD8^+^ T cells, immunity, SARS‐CoV‐2, vaccination

## Abstract

Effective vaccines have reduced the morbidity and mortality caused by severe acute respiratory syndrome coronavirus‐2 infection; however, the elderly remain the most at risk. Understanding how vaccines generate protective immunity and how these mechanisms change with age is key for informing future vaccine design. Cytotoxic CD8^+^ T cells are important for killing virally infected cells, and vaccines that induce antigen‐specific CD8^+^ T cells in addition to humoral immunity provide an extra layer of immune protection. This is particularly important in cases where antibody titers are suboptimal, as can occur in older individuals. Here, we show that in aged mice, spike epitope–specific CD8^+^ T cells are generated in comparable numbers to younger animals after ChAdOx1 nCoV‐19 vaccination, although phenotypic differences exist. This demonstrates that ChAdOx1 nCoV‐19 elicits a good CD8^+^ T‐cell response in older bodies, but that typical age‐associated features are evident on these vaccine reactive T cells.

## Graphical abstract

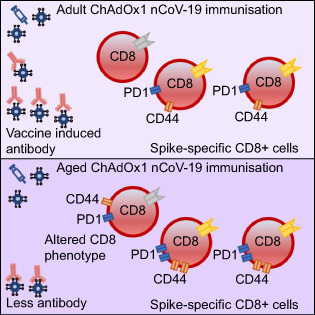

## INTRODUCTION

Since the beginning of the severe acute respiratory syndrome coronavirus‐2 (SARS‐CoV‐2) pandemic in 2020, multiple effective vaccines have been produced. This includes the ChAdOx1 nCoV‐19 vaccine (AZD1222), which has been supplied in billions of doses worldwide. The ChAdOx1 nCoV‐19 vaccine is an adenovirus‐vectored vaccine, which can stimulate CD8^+^ T‐cell responses as well as inducing neutralizing antibodies.^1^, ^2^ ChAdOx1 nCoV‐19 can prevent symptomatic infections, limit viral transmissibility, reduce infection severity and prevent hospitalizations and death caused by variants of concern.^3^, ^4^, ^5^, ^6^ Vaccine efficacy is associated with the effective generation of anti‐spike protein antibodies.^7^ While generation of protective humoral immunity is a key function of vaccines, adenovirus vector vaccine–induced memory CD8^+^ T cells also provide cellular immunity.^8^, ^9^ Murine experiments using protein‐based vaccines and challenge with SARS‐CoV‐2 variants showed that, in the absence of viral‐neutralizing antibodies, CD8^+^ T cells provide protection, with depletion of CD8^+^ T cells leading to an increase in viral load.^10^ Furthermore, CD8^+^ T cells can provide cross‐protective immunity to variants of concern, across multiple vaccine platforms, providing protection as the virus continues to evolve.^11^

In general, aging negatively impacts the function of immune system, and for coronavirus disease 2019 (COVID‐19) infections, likely contributes to the strong link between advancing age and risk of hospitalization or death.^12^ Moreover, vaccination becomes less effective with increased age, as older individuals have lower serum neutralization and immunoglobulin (Ig)G/A titers after a single vaccination with Pfizer's BNT162b2 messenger RNA vaccine.^13^ Furthermore, older patients have responses that wane more quickly, meaning increased risk over time.^14^ Despite this, the phase 3 trial of ChAdOx1 nCoV‐19 found that vaccine efficacy was maintained in participants over 65 years of age, despite lower humoral immunity.^15^ During aging, the CD8^+^ T‐cell compartment changes; thymic involution occurs in early adulthood and reduces the supply of new naïve T cells, and later, an accumulation of differentiated cells with a central memory (CD44^+^ CD62L^+^) phenotype occurs, although these cells may include antigen‐independent differentiation of virtual memory T cells.^16^ Age‐associated effector subset expansion is more pronounced in CD8^+^ T cells than in CD4^+^ T cells, with CD8^+^ T cells exhibiting lesser homeostatic stability^17^; naïve CD8^+^ T‐cell frequency declines more drastically over time, as clones are either lost or differentiate into central/virtual memory cells.^18^, ^19^

Here, we aimed to characterize the production of antigen‐specific CD8^+^ T cells following a single ChAdOx1 nCoV‐19 vaccination, to understand how their number and phenotype change in aging. We report that while aged mice have decreased humoral immunity following a single ChAdOx1 nCoV‐19 vaccination, they successfully generate comparable numbers of spike‐specific CD8^+^ T cells to younger adult animals, albeit with an altered phenotype.

## RESULTS

### Aged mice have reduced serum humoral immunity after ChAdOx1 nCoV‐19 vaccination

**Figure 1:**
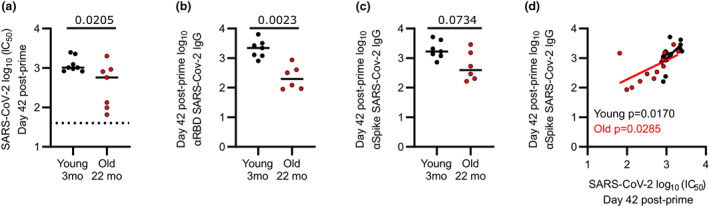
Advanced age negatively affects serum immunity after ChAdOx1 nCoV‐19 vaccination. Young (3 months of age) and old (22 months of age) mice were immunized with 50 μL ChAdOx1 nCoV‐19 (10^8^ infectious units) intramuscularly. At 42 days after vaccination serum samples were analyzed. **(a)** Severe acute respiratory syndrome coronavirus‐2 (SARS‐CoV‐2) serum neutralizing capacity expressed as reciprocal serum dilution required to inhibit pseudotyped virus entry by 50% (IC_50_). The dotted line represents lower detection limits. **(b, c)** Serum anti‐RBD **(b)** and anti‐spike **(c)** immunoglobulin (Ig)G antibodies. **(d)** IC_50_ *versus* anti‐spike IgG serum antibody. **(a**) Young mice, *n* = 8; old mice *n* = 7. **(b, c)** Young mice, *n* = 7; old mice *n* = 6. Data are representative of two individual experiments. **(d)** Young mice, *n* = 15; old mice *n* = 13. Data are combined from two individual experiments. **(a–c)** Mann–Whitney *U*‐tests were used. **(d)** Separate linear regressions were performed for young and old mice, respectively. mo, month old; RBD, receptor‐binding domain.

Aging is known to impact humoral immunity after vaccination. To test whether the CD8^+^ T‐cell response is intact in aging despite humoral immunity being reduced, we first determined the total and neutralizing antibody titer after ChAdOx1 nCoV‐19 vaccination. Indeed, 42 days after intramuscular vaccination, 22‐month‐old mice had lower serum SARS‐CoV‐2 pseudovirus neutralizing capacity than 3‐month‐old mice (Figure 1a). Consistent with this, 22‐month‐old mice also had a reduction in both receptor‐binding domain (RBD; Figure 1b) and spike (Figure 1c) binding IgG in the serum. We also correlated these two metrics of vaccine protection, showing that mice with higher neutralizing capacity had higher serum spike‐specific antibody (Figure 1d), indicating that titer rather than quality of the antibody response is impaired with advanced age. These results confirm previous findings that aging negatively affects the generation of antibody following prime vaccination for SARS‐CoV‐2.^2^, ^13^, ^20^

### Aged mice generate comparable numbers of spike‐specific CD8^+^ T cells after ChAdOx1 nCoV‐19 vaccination

**Figure 2:**
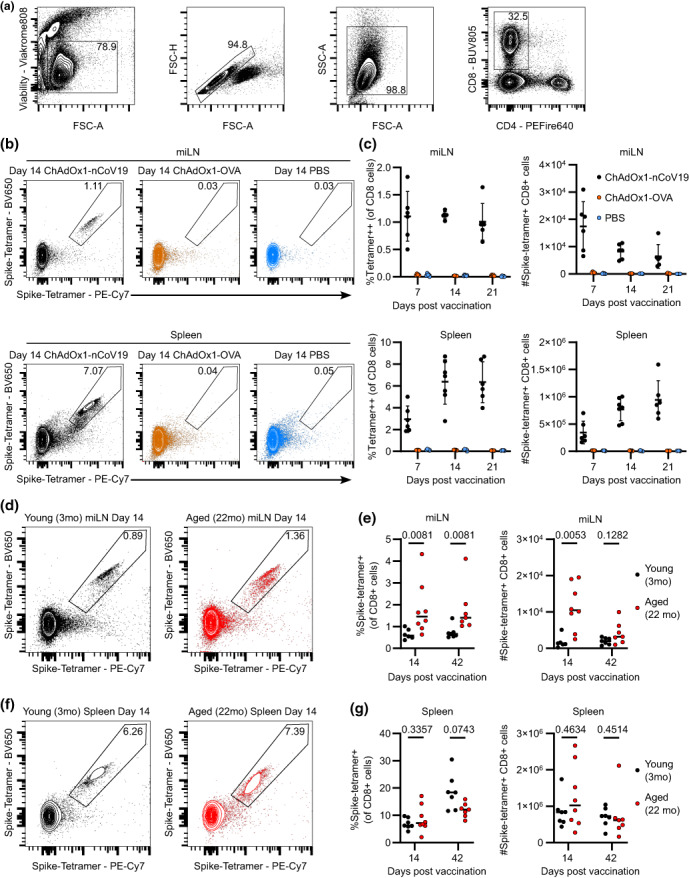
Aged mice generate comparable numbers of spike‐specific CD8^+^ T cells after ChAdOx1 nCoV‐19 vaccination. Mice were immunized with 50 μL of ChAdOx1 nCoV‐19, ChAdOx1 ovalbumin (10^8^ infectious units) or phosphate‐buffered saline intramuscularly. At the indicated timepoints, medial iliac lymph node (miLN) and spleen samples were taken for analysis. **(a)** Live, single, CD8^+^ T cells were identified—representative plots from miLN, 14 days after vaccination. **(b)** miLN and spleen spike protein tetramer (SARS‐CoV‐2 S 539–546—VNFNFNGL) binding CD8^+^ T cells were identified by flow cytometry. **(c)** Frequency (as a percentage of all CD8^+^ T cells) and number of spike‐protein‐specific CD8^+^ T cells was quantified in miLN and spleen. **(d)** Spleen spike protein binding CD8^+^ T cells were identified in both young and old mice. **(e)** Frequency (as a percentage of all CD8^+^ T cells) and the number of spike‐protein–specific CD8^+^ miLN T cells were quantified in 3‐ and 22‐month‐old mice at the indicated timepoints. **(f)** Spleen spike protein–binding CD8^+^ T cells were in both young and old mice. **(g)** Frequency (as a percentage of all CD8^+^ T cells) and number of spike‐protein–specific spleen CD8^+^ T cells were quantified in 3‐ and 22‐month‐old mice at the indicated timepoints. Each symbol represents a unique biological sample. Data are representative of two individual experiments. **(e, g)** Multiple Mann–Whitney *U*‐tests per row with multiple testing correction was used. SARS‐CoV‐2, severe acute respiratory syndrome coronavirus‐2. FSC‐A, forward scatter–area; FSC‐H, forward scatter–height; mo, months old; PBS, phosphate‐buffered saline; PE, phycoerythrin.

To track spike‐specific CD8^+^ T cells directly *ex vivo* after vaccination, we used a major histocompatibility complex (MHC) class I peptide tetramer (SARS‐CoV‐2 S 539–546—VNFNFNGL) to detect spike‐binding CD8^+^ T cells in the draining medial iliac lymph node (mILN) and spleen of 3‐month‐old mice (Figure 2a, b). We validated the specificity of the tetramer in mice immunized with ChAdOx1‐ovalbumin or those which received a saline‐only injection (Figure 2b, c). Typically, after vaccination, the draining lymph node has a higher frequency of antigen‐specific B or CD4^+^ T cells, than the spleen.^2^ However, we found that the spleen has a greater frequency of antigen‐specific CD8^+^ T cells following ChAdOx1 nCoV‐19 vaccination than the draining mILN and is therefore the primary reservoir of these cells (Figure 2b, c). We next sought to understand the effect of aging on the spike‐specific CD8 T‐cell response, and therefore we vaccinated young (3 months of age) and aged (22 months of age) mice (Figure 2d). We found that the 22‐month‐old mice had an increased frequency of spike‐specific CD8^+^ T cells at both 14‐ and 42‐day following vaccination, which corresponded to a greater number of spike‐specific CD8^+^ T cells in the mILN at day 14 (Figure 2e). In the spleen, there was no difference in either the frequency or the total number of spike‐specific CD8^+^ T cells between 3‐ and 22‐month‐old mice at either 14‐ or 42‐day following vaccination (Figure 2f, g). Thus, despite the various age‐associated defects in lymphocyte biology following vaccination, expansion of spike‐specific CD8^+^ T cells occurs effectively in 22‐month‐old mice in response to ChAdOx1 nCoV‐19 and these cells are maintained for at least 42 days.

### Spike protein–specific CD8^+^ T cells from aged mice have an altered phenotype

**Figure 3:**
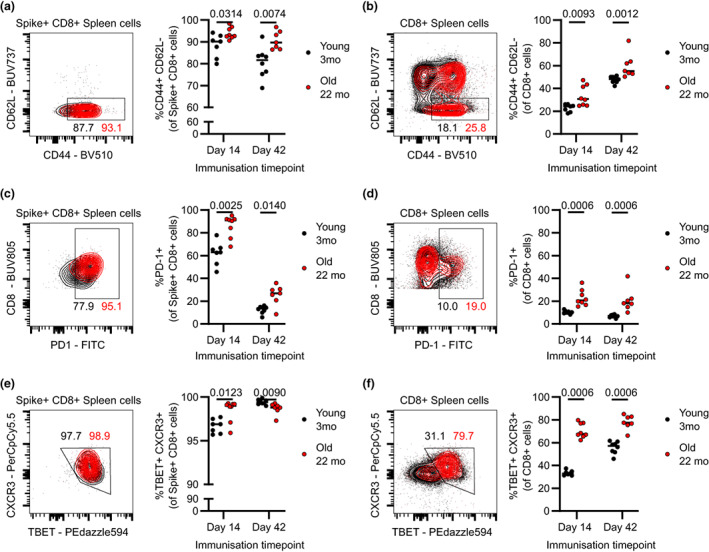
Splenic spike‐protein–specific CD8^+^ T cells from aged mice have increased expression of activation and exhaustion markers. Mice were immunized with 50 μL of ChAdOx1 nCoV‐19 (10^8^ infectious units) intramuscularly. At the indicated timepoints, spleen samples from 3‐ and 22‐month‐old mice were taken to analyze the frequency of various CD8^+^ T‐cell subsets by flow cytometry. **(a)** Spike‐specific CD44^+^ CD62L^−^ (T‐effector memory) phenotype. **(b)** Total CD44^+^ CD62L^−^ (T effector) phenotype. **(c)** Spike‐specific programmed cell death protein 1 (PD‐1) expressers. **(d)** Total PD‐1 expressers. **(e)** Spike‐specific T‐BET^+^ CXCR3^+^ (activated) phenotype. **(f)** Total T‐BET^+^ CXCR3^+^ (activated) phenotype. Each symbol represents a unique biological sample. Data are representative of two individual experiments. **(a–f)** Multiple Mann–Whitney *U*‐tests per row with multiple testing correction were used. FITC, fluorescein isothiocyanate; CXCR3, C–X–C chemokine receptor type 3; PD‐1, programmed cell death protein 1.

Following the observation that old mice generated spike‐specific CD8^+^ T cells effectively, we next explored their phenotype. Given that the spike‐specific CD8^+^ T cells in the spleen outnumbered those of the mILN by a factor of about 100, we first analyzed the spleen. We sought to contextualize our observations by performing a side‐by‐side comparison of the spike‐specific CD8^+^ T‐cell population alongside total CD8^+^ T cells in the vaccinated mice. The majority of spike^+^ CD8^+^ T cells were of CD44^+^ CD62L^−^ T‐effector (Teff) phenotype that marks both effector and effector memory T cells, and 22‐month‐old mice had a near ubiquitous Teff phenotype within their spike^+^ CD8^+^ T cells (Figure 3a). For the total CD8 cell population, 22‐month‐old mice had a higher frequency of CD8^+^ T cells with Teff phenotype, at both timepoints after vaccination (Figure 3b). Programmed cell death protein 1 (PD‐1) can be a marker of T‐cell activation, and consistent with this, spike‐specific CD8^+^ T cells from mice of both age groups had a high frequency of PD‐1 expression 14‐day after vaccination, which dropped after 42 days, with spike‐specific CD8^+^ T cells from 22‐month‐old mice retaining higher PD‐1 expression at this later timepoint than those from 3‐month‐old mice (Figure 3c). This trend was also seen across the entire CD8^+^ T‐cell pool, in which 22‐month‐old mice had more PD‐1^+^ cells than young mice (Figure 3d). Expression of T‐BET and C–X–C chemokine receptor type 3 (CXCR3) in antigen‐specific CD8^+^ T cells is associated with an activated effector phenotype.^21^, ^22^ We quantified the expression of these markers in our study and found that spike^+^ CD8^+^ T cells for both 3‐ and 22‐month‐old mice were more than 95% positive for these markers, with subtle differences between age groups (Figure 3e). Across all CD8^+^ T cells, 22‐month‐old mice had an increased frequency of T‐BET^+^ CXCR3^+^ dual expressing cells, suggesting an overall skew toward an activated phenotype (Figure 3f).

**Figure 4:**
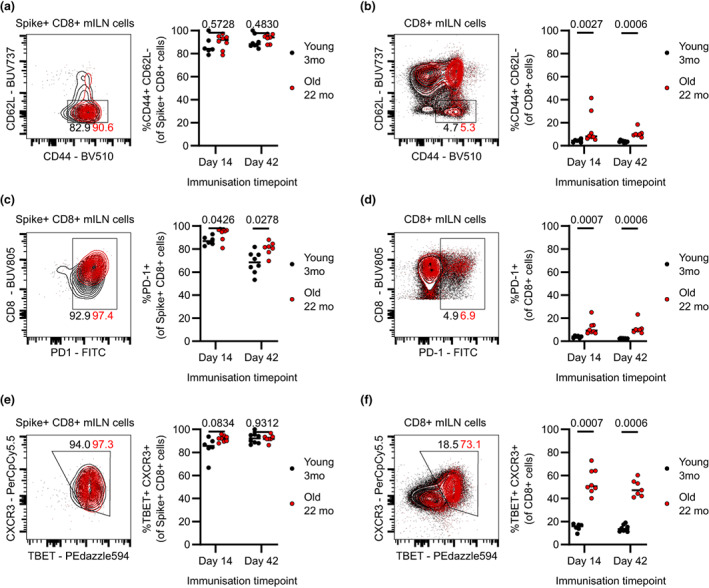
Medial iliac lymph node (mILN) spike‐protein–specific CD8^+^ T cells from aged mice have increased expression of activation and exhaustion markers. Mice were immunized with 50 μL of ChAdOx1 nCoV‐19 (10^8^ infectious units) intramuscularly. At the indicated timepoints, mILN samples from 3‐ and 22‐month‐old mice were taken to analyze the frequency of various CD8^+^ T‐cell subsets by flow cytometry. **(a)** Spike‐specific CD44^+^ CD62L^−^ (T‐effector memory) phenotype. **(b)** Total CD44^+^ CD62L^−^ (T effector) phenotype. **(c)** Spike‐specific PD‐1 expressers. **(d)** Total PD‐1 expressers. **(e)** Spike‐specific T‐BET^+^ CXCR3^+^ (activated) phenotype. **(f)** Total T‐BET^+^ CXCR3^+^ (activated) phenotype. Each symbol represents a unique biological sample. Data are representative of two individual experiments. **(a–f)** Multiple Mann–Whitney *U*‐tests per row with multiple testing correction were used. CXCR3, C–X–C chemokine receptor type 3; FITC, fluorescein isothiocyanate; PD‐1, programmed cell death protein 1.

These splenic observations were largely recapitulated in the mILN, as nearly all spike‐specific cells had a Teff phenotype (Figure 4a), while 22‐month‐old mice had an overall increased frequency of Teff phenotype CD8^+^ T cells (Figure 4b). We also noted that Teff phenotype cells were less frequent in the mILN than in the spleen (Figures 3b and 4b). Like the spleen, 22‐month‐old mice had an increased frequency of PD‐1 expression in both spike‐specific and total CD8^+^ cells 14‐day after vaccination, with spike‐specific CD8^+^ T cells largely maintaining their PD‐1 expression 42 days after vaccination (Figure 4c, d). Again, spike‐specific mILN CD8^+^ cells were near ubiquitously T‐BET and CXCR3 double‐positive (Figure 4e). Finally, total CD8^+^ cells from 22‐month‐old mice had increased frequency of T‐BET and CXCR3 coexpression as compared with 3‐month‐old mice (Figure 4f). Thus, aging is associated with the increased acquisition of activation markers associated with effector phenotype on CD8^+^ T cells, with these markers being very frequently expressed by spike‐specific CD8^+^ T cells.

## DISCUSSION

In this study, we compared the response made by young and old mice following ChAdOx1 nCoV‐19 vaccination, showing that while humoral immunity is compromised in aging, spike‐epitope–specific CD8^+^ T cells still expand, albeit with an altered phenotype. Six weeks after vaccination, serum from 22‐month‐old mice had reduced SARS‐CoV‐2 pseudoneutralizing capacity, as well as lower spike‐specific IgG titer. This agrees with our previous findings, as well as findings of many others that humoral immunity is compromised in advanced age.^2^, ^13^, ^23^ Despite this reduction in humoral immunity, we found that expansion of spike‐specific CD8^+^ T cells occurs effectively, and is potentially enhanced in aged mice, with the spleen acting as the primary reservoir in which millions of spike‐specific CD8^+^ T cells can be found. In aged mice, however, there is a shift of phenotype among CD8^+^ T cells after vaccination, and this is seen to an even greater extent within the recently activated spike epitope–specific pool of CD8^+^ T cells evaluated here, with increased expression of PD‐1 and CD44 and dual expression of T‐BET and CXCR3.

A known feature of the aging immune system is a reduction of T‐cell receptor repertoire diversity, which occurs as a result of thymic involution and expansion of antigen‐experienced clones.^24^ This loss of CD8^+^ T‐cell diversity is nonrandom, being at least in part driven by T‐cell receptor:pMHC avidity, such that cells specific for epitopes with poor self‐pMHC avidity are likely to be lost.^25^ This therefore impacts the response to specific antigens; for example, following influenza infection aged mice have a reduced response to the immunodominant nucleoprotein epitope NP_366–374_.^26^ Our results suggest that clones specific for spike epitopes are maintained in aged mice, such that antigen‐specific CD8^+^ T cells are successfully generated following ChAdOx1 nCoV‐19 vaccination, but produce fewer effector cytokines following spike peptide‐pool stimulation, despite their activated phenotype.^2^

As aging occurs, the microenvironment becomes more proinflammatory.^27^ This increased concentration of inflammatory cytokines and resultant low‐level signaling likely contributes to an altered baseline phenotype and leaves CD8^+^ T cells less capable to respond to stimuli.^28^ A continual supply of low‐grade activation signals results in differentiation into a virtual memory state, the development of which correlates with the age‐related decline seen in primary responses.

Virtual memory cells can be produced from naïve T cells undergoing interaction with self‐antigen. These cells can then provide protective immunity in a nonantigen‐specific manner, being recruited into secondary lymphoid organs during active immune responses by cues such as CXCR3 signaling and providing bystander killing in an interleukin‐15–dependent manner.^29^, ^30^ The virtual memory CD8^+^ T‐cell pool that forms in aged mice may indeed contain cells which are spike specific, and thus may contain the progenitors of the spike‐specific CD8^+^ T cells we describe here, although we were not able to establish this experimentally. The age‐associated rise of virtual memory CD8^+^ T cells may counteract some elements of immunosenescence, as virtual memory cells maintain the ability to undergo asymmetric cell division and expand efficiently.^31^ However, despite expanding well, virtual memory cells have limited functionality and become senescent with age, and transfer of old CD8^+^ T cells into young mice does not rescue their responsiveness, potentially because of epigenetic imprinting.^32^, ^33^ A potential virtual memory origin of these cells may suggest that while spike‐specific CD8^+^ T cells expand, they could lose their functional capacity—producing fewer cytokines upon restimulation.^2^ In our previous study,^2^ restimulation of bulk splenic CD8^+^ T cells with SARS‐CoV‐2 peptide pools from ChAdOx1 nCoV‐19–vaccinated mice resulted in a nonstatistically significant trend to fewer cytokine‐secreting CD8 cells in aged mice. Importantly, this trend was not observed once the mice received a second ChAdOx1‐nCoV19 booster immunization.

Therapeutics aiming to reverse T‐cell exhaustion by targeting PD‐1 are becoming popular, and a recent paper highlighted that patients with cancer treated with anti‐PD‐1 immunotherapy had an increase in proliferation within their PD‐1 expressing CD4^+^ T follicular helper cells.^34^ Short‐term anti‐PD‐1 treatment has been shown to increase the protection provided by simian immunodeficiency virus vaccines in macaques and increases viral clearance and CD8^+^ memory precursor formation during lymphocytic choriomeningitis virus infection of mice.^35^, ^36^ Given the increased expression of PD‐1 in aged mice shown here, targeting this pathway during SARS‐CoV‐2 vaccination may boost CD8^+^ T‐cell responses, although the risk of self‐reactive T‐cell development may limit its use as a vaccine enhancing strategy.

In summary, we show that the ChAdOx1 nCoV‐19 vaccine successfully expands spike peptide–specific CD8^+^ T cells in both younger adult and aged mice, agreeing with recent data showing that despite acquiring exhaustion markers such as PD‐1, CD8^+^ T cells are capable of continued supernumerary cell division in the appropriate context,^37^ although CD8^+^ T cells from aged mice produce fewer cytokines than their younger counterparts when directly stimulated with spike protein peptides.^2^ This suggests that for future vaccines to successfully generate cytotoxic CD8^+^ T‐cell immunity in aged individuals, they must target epitopes that survive the age‐associated contraction of T‐cell repertoire, and that the background environment into which the vaccine is introduced plays an unavoidably important role for CD8^+^ T‐cell responses.

Adenovirus‐based vaccines are an attractive option for vaccinating the elderly, as ChAdOx1 nCoV‐19 vaccination successfully expands antigen‐specific CD8^+^ T cells in aged mice, and this layer of cellular immunity is maintained to a greater extent than humoral immunity when viral variants occur.^38^

## METHODS

### Mouse housing, husbandry and immunization

C57BL/6Babr mice were bred and maintained in the Babraham Institute Biological Support Unit. No primary pathogens or additional agents listed in the Federation of European Laboratory Animal Science Associations (FELASA) recommendations were detected during health monitoring surveys of the stock holding rooms. Ambient temperature was ≈19–21°C and relative humidity 52%. Lighting was provided on a 12‐h light:12‐h dark cycle including 15‐min “dawn” and “dusk” periods of subdued lighting. After weaning, mice were transferred to individually ventilated cages with up to five mice per cage. Mice were fed CRM (P) VP diet (Special Diets Services, Augy, France) *ad libitum* and received seeds at the time of cage cleaning as part of their environmental enrichment. All mouse experimentation was approved by the Babraham Institute Animal Welfare and Ethical Review Body. Animal husbandry and experimentation complied with existing European Union and United Kingdom Home Office legislation and local standards (PPL: P4D4AF812). Mice were immunized at 10–12 weeks of age and 95–101 weeks of age for young and old groups, respectively. Mice were immunized in the right quadriceps femoris muscle with 10^8^ infectious units of ChAdOx1 nCoV‐19 or ChAdOx1 ovalbumin in 50 μL phosphate‐buffered saline.

### Microneutralization test using lentiviral‐based pseudotypes bearing the SARS‐CoV‐2 spike

Lentiviral‐based SARS‐CoV‐2 pseudotyped viral particles were generated in HEK293T cells as previously described.^39^ Cells were seeded in 6‐well dishes, before being transfected with SARS‐CoV‐2 spike, p8.91 (encoding HIV‐1 gag‐pol) and CSFLW in Opti‐MEM (Thermo Fisher Scientific, Waltham, MA, USA) along with 10 μL polyethylenimine transfection reagent overnight. Then, the transfection mix was replaced with 3 mL Dulbecco's Modified Eagle Medium (Thermo Fisher Scientific) with 10% fetal bovine serum (Thermo Fisher Scientific) and incubated for 48 and 72 h, and then SARS‐CoV‐2 pps supernatants were pooled and centrifuged to remove cellular debris. Target HEK293T cells (transfected with human ACE2 expression plasmid) were seeded at a density of 2 × 10^4^ in 100 μL Dulbecco's Modified Eagle Medium‐10% overnight. SARS‐CoV‐2 pseudotyped viral particles were titrated 10‐fold on target cells. Sera were diluted 1:20 in serum‐free media and added to a 96‐well plate in triplicate and titrated twofold. A fixed titered volume of SARS‐CoV‐2 pseudotyped viral particles were added at a dilution equivalent to 10^5^ signal luciferase units in 50 μL Dulbecco's Modified Eagle Medium‐10% and incubated with sera for 1 h. Target cells expressing human ACE2 were then added at a density of 2 × 10^4^ in 100 μL and incubated for 72 h. Firefly luciferase activity was then measured with Bright‐Glo luciferase reagent and a Glomax‐Multi+ Detection System (Promega, Southampton, UK).

### ELISA

Standardized ELISA was performed to detect SARS‐CoV‐2 spike or RBD‐specific antibodies. MaxiSorp plates (Thermo Fisher Scientific) were coated with 100 ng/well protein overnight at 4°C. Plates were washed with phosphate‐buffered saline + 0.05% Tween‐20 and blocked with Blocker Casein in phosphate‐buffered saline (Thermo Fisher Scientific) for 1 h at room temperature. Sera (including positive, negative and internal control samples) diluted in casein were incubated for 2 h at room temperature. Plates were washed, then alkaline phosphatase‐conjugated goat anti‐mouse IgG or alkaline phosphatase‐conjugated goat anti‐human IgG (Sigma‐Aldrich, St. Louis, MO, USA) with pNPP substrate (Sigma‐Aldrich) was added for 1 h at room temperature. An arbitrary number of ELISA units were assigned to the positive control samples and optical density values of each dilution were fitted to a four‐parameter logistic curve using SOFTmax PRO software (Molecular Devices, San Jose, CA, USA). Sample ELISA values were calculated using the generated standard curve.

### MHC‐1 tetramer generation and flow cytometry

MHC‐1 monomers were acquired from the NIH tetramer core (Emory, GA, USA), and conjugated to streptavidin following provided instructions. A 4:1 molar ratio was calculated for monomer:streptavidin incubation. Streptavidin was added to monomers in 10% increments, with each 10% added after 10 min, at room temperature in the dark. Once completely combined, reagents were left overnight at 4°C before use.

mILNs/spleens were pressed through a 70‐μm mesh and washed through with flow cytometry staining buffer (phosphate‐buffered saline containing 2% fetal bovine serum and 1 mM ethylenediaminetetraacetic acid) to generate single‐cell suspensions. Cell numbers and viability were determined using a CASY TT Cell Counter (Roche, Basel, Switzerland). About 2 × 10^6^ cells were transferred into 96‐well V‐bottomed plates, which were then centrifuged and the supernatant was removed. Cells were resuspended in 50 μL Roswell Park Memorial Institute medium containing 200 nM dasatinib (added to prevent T‐cell receptor internalization). Cells were incubated for 15 min at 37°C, before 50 μL Roswell Park Memorial Institute medium + dasatinib containing MHC‐1 spike–specific tetramers were added. Cells were then incubated for 105 min at room temperature in the dark. Cells were washed with FACS buffer and stained with 100 μL surface antibody mix (including viability dye) for 2 h at 4°C. Cells were then fixed with eBioscience Foxp3/Transcription factor fixation kit (Thermo Fisher Scientific). Cells were washed two times with FACS buffer and fixed with fixation reagent diluted as per manufacturer's instructions for 30 min at 4°C. Cells were then washed with 1× permeabilization buffer two times and stained with intracellular antibody mix in permeabilization buffer supplemented with 20% 2.4G2 hybridoma (ATCC hb‐197) (American Type Culture Collection, Manassas, VA, USA) tissue culture supernatant at 4°C overnight. Cells were washed two times with permeabilization buffer and once with FACS buffer and acquired on a 5‐laser Cytek Aurora spectral flow cytometer (Cytek, Fremont, CA, USA). Cells for single‐color controls were prepared in the same manner as fully stained samples. The antibodies used for surface and overnight staining are listed below. Manual gating of flow cytometry data was done using FlowJo version 10.7 software (FlowJo LLC, Ashland, OH, USA). Antibodies used are listed in Table 1.

**Table 1:** The list of antibodies used.

Antibody/Fluorophore	Supplier	Catalog number	RRID
Brilliant Violet 510 anti‐mouse/human CD44 antibody	BioLegend	103 044	AB_2650923
PE/Dazzle 594 anti‐T‐bet antibody	BioLegend	644 828	AB_2565677
BUV737 Rat Anti‐Mouse CD62L	BD Biosciences	612 833	AB_2870155
PerCP/Cyanine5.5 anti‐mouse CD183 (CXCR3) antibody	BioLegend	126 514	AB_1186017
CD279 (PD‐1) monoclonal antibody (RMP1‐30), FITC	Thermo Fisher Scientific	11–9981‐82	AB_465467
PE/Fire 640 anti‐mouse CD4 antibody	BioLegend	100 482	AB_2860585
BUV805 rat anti‐mouse CD8a	BD Biosciences	564 920	AB_2716856
Brilliant Violet 650 streptavidin	BioLegend	405 232	
PE/Cyanine7 streptavidin	BioLegend	405 206	

PD‐1, programmed cell death protein 1.

## AUTHOR CONTRIBUTIONS

**William S Foster:** Conceptualization; data curation; formal analysis; investigation; methodology; project administration; visualization; writing – original draft; writing – review and editing. **Nazia Thakur:** Investigation; methodology; writing – review and editing. **Joseph Newman:** Investigation; methodology; writing – review and editing. **Alexandra J Spencer:** Investigation; methodology; writing – review and editing. **Sophie Davies:** Investigation; methodology; writing – review and editing. **Danielle Woods:** Investigation; methodology; writing – review and editing. **Leila Godfrey:** Investigation; methodology; writing – review and editing. **Sarah H Ross:** Methodology; supervision; writing – review and editing. **Arianne C Richard:** Methodology; supervision; writing – review and editing. **Hayley J Sharpe:** Methodology; supervision; writing – review and editing. **Dalan Bailey:** Project administration; supervision; writing – review and editing. **Teresa Lambe:** Conceptualization; funding acquisition; resources; supervision; writing – review and editing. **Michelle A Linterman:** Conceptualization; funding acquisition; project administration; supervision; writing – original draft; writing – review and editing.

## CONFLICT OF INTEREST

TL is named on a patent application covering ChAdOx1 nCoV‐19. The remaining authors declare no competing interests. The funders played no role in the conceptualization, design, data collection, analysis, decision to publish or preparation of the manuscript. AJS, SD, DW, LG and TL are contributors to intellectual property licensed by Oxford University Innovation to AstraZeneca.

## FUNDING INFORMATION

This study was supported by funding from the Biotechnology and Biological Sciences Research Council (BBS/E/B/000C0427, BBS/E/B/000C0428 and the Campus Capability Core Grant to the Babraham Institute), the Lister institute of Preventative Medicine, the EPSRC VaxHub (EP/RO13756/1) and Innovate UK (biEBOV: 971615). TL and AJS are Jenner Investigators. HJS is supported by a Sir Henry Dale Fellowship jointly funded by the Wellcome Trust and the Royal Society [109407] and a BBSRC institutional programme grant [BBS/E/B/000C0433]. MAL is an EMBO Young Investigator and Lister Institute Prize Fellow. NT, JN, ACR and DB were supported by the MRC (MR/W005611/1, MR/W016303/1) and BBSRC (BBS/E/I/COV07001, BBS/E/I/00007031 and BB/T008784/1).

## Data Availability

The data that support the findings of this study are available from the corresponding author upon reasonable request.
